# Impact of Thymidine Loop Modifications on Telomeric G-Quadruplex Catalytic Systems for Asymmetric Sulfoxidation

**DOI:** 10.3390/molecules31030442

**Published:** 2026-01-27

**Authors:** Claudia Finamore, Carmen Festa, Daniela Benigno, Carla Aliberti, Rosa Barbato, Simona De Marino, Aldo Galeone, Veronica Esposito, Antonella Virgilio

**Affiliations:** Department of Pharmacy, University of Naples Federico II, Via D. Montesano 49, I-80131 Naples, Italy; claudia.finamore@unina.it (C.F.); carmen.festa@unina.it (C.F.); daniela.benigno@unina.it (D.B.); carla.aliberti@unina.it (C.A.); rosa.barbato3@unina.it (R.B.); sidemari@unina.it (S.D.M.); galeone@unina.it (A.G.)

**Keywords:** G-quadruplex, sulfoxidation, DNAzymes, asymmetric synthesis

## Abstract

G-quadruplex (G4) DNA structures have recently emerged as promising chiral scaffolds for enantioselective catalysis. This study investigates how thymidine loop modifications influence the catalytic performance of the telomeric G4 sequence HT21 in the asymmetric sulfoxidation of thioanisole. To this end, several singly or doubly modified HT21 derivatives were synthesized by using β-L-2′-deoxythymidine, 5-hydroxymethyl-2′-deoxyuridine, and 5-bromo-2′-deoxyuridine instead of a T residue, or β-L-2′-deoxyadonesine instead of an A residue, in specific positions within the TTA loops. The catalytic activity of these analogues was evaluated in the Cu(II)-mediated oxidation of thioanisole using hydrogen peroxide as oxidant. All modified sequences maintained complete substrate conversion, but their enantioselectivities varied markedly. Whereas the highest enantiomeric excess (84% ee) had previously been achieved with the HT21 analogue bearing a β-L-2′-deoxyadenosine in the first loop, the thymidine-based modifications, either alone or in combination, resulted in lower ee values, suggesting that loop alterations critically affect the chiral microenvironment, not all loop positions are functionally equivalent, and single substitutions within the same loop can result in different enantioselectivities. These findings highlight new insights on how individual loop residues contribute to asymmetric induction and offer further details for tuning G4-based catalytic scaffolds.

## 1. Introduction

The need for chiral molecules in various fields such as materials science, agrochemicals, and pharmaceuticals is growing year after year, driving the development of enantioselective catalytic processes which now represent milestones of modern synthetic chemistry [1,2,3]. Chirality is a central property in molecular chemistry, representing a key aspect in a plethora of scientific and industrial fields. The distinct configurations of chiral molecules can engage in different molecular interactions; therefore, in pharmaceutical applications, the specific spatial orientation of these molecules can significantly influence both the efficacy and the safety of drugs. The implications of chirality are crucial not only in medicine but also in the food industry, affecting flavors and aromas which are critical for user taste and product success, and in agriculture where they act as chiral pesticides selective only for targeted pests, raising crop safety and decreasing environmental poisonousness.

Although the chemical industry makes extensive use of several methods for chiral catalysis, such as resolution or using chiral pool reagents, an interesting approach to achieving enantiomerically pure compounds is asymmetric synthesis employing chiral catalysts to control the stereochemistry of the reaction. 

Generally, the chiral catalyst interacts with the substrate by enveloping it in a chiral environment that favors the formation of one enantiomer over the other. One of the most successful strategies for enantioselective synthesis is based on isolated enzymes as biocatalysts, reagents capable of guaranteeing high enantiomeric excesses and specificity, as well as being environmentally benign. Enzymes exhibit extreme selectivity, particularly enantioselectivity, towards their substrates, since they are composed of L-amino acids, which make the enzyme chiral—allowing it to transfer its chirality to the substrate and forming the different possible stereoisomers in diverse quantities [4]. However, when enzymes are applied as biocatalysts on non-natural substrates, they often exhibit low activity and enantioselectivity, thus sometimes requiring complex modifications to enhance their stability, activity, and selectivity [5,6]. 

Recently, the discovery of DNA-based catalysts has opened new perspectives for asymmetric synthesis. DNAzymes, or catalytic DNA, compared with protein enzymes are generally more stable and easier to synthesize. Moreover, DNAzymes can be straightforwardly modified to catalyze different reactions, offering better catalytic activity than other natural enzymes. Therefore, DNAzymes have important applications in several biochemical fields, including biosensing and biomedicine [7,8]. 

Among the possible catalytically active secondary structures adopted by DNAzymes, G-quadruplexes (G4s) have arisen as hopeful scaffolds for enantioselective synthesis. These nucleic acid structures are formed by G-rich sequences that can fold into stable four-stranded conformations through π–π stacking of planar G-quartets, which are themselves stabilized by Hoogsteen hydrogen bonds [9]. G4 DNA can adopt multiple conformations, such as antiparallel, parallel, and hybrid structures [10]. Due to their unique topology, structural variability, and ability to interact with various cofactors, they can provide several advantages for achieving high enantiomeric excesses in different enantioselective reactions. Among these transformations, sulfoxidation is of particular interest in the pharmaceutical field. Sulfoxides, owing to their diverse bioactivities, are highly attractive scaffolds in drug design, and many clinically used compounds contain chiral sulfoxides whose enantiomeric forms can display markedly different biological properties [11]. This underscores the need for asymmetric oxidation methods to prepare individual enantiopure sulfoxides, since in most cases they are obtained from the corresponding sulfides through oxidation in the presence of suitable catalysts [12,13,14,15]. 

In this framework, G4 DNA catalysts represent an interesting alternative as biocatalysts. Several studies have revealed that G4s, associated with appropriate cofactors, such as hemin or transition metal complexes, can catalyze sulfoxidation with notable enantiomeric excess (ee), thus indicating that diverse G4 DNA conformations provide a straightforward tool for designing enantioselective DNA hybrid catalysts [16]. It is known that various metal complexes target G4 DNA by stacking on G-tetrads [17]. Therefore, G4 DNA hybrid catalysts can be self-assembled based on non-covalent interaction between G4 DNA and metal complexes. 

**Figure 1:**
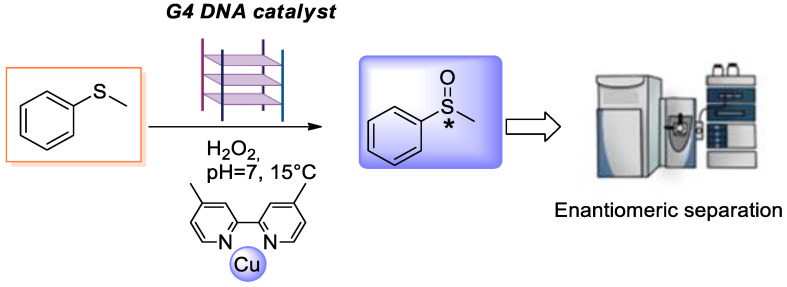
Sulfoxidation by a dimethyl bipyridyl–copper(II) complexas reported by Cheng et al. [18]. The asterisk (*) indicates a stereogenic (chiral) center.

According to this strategy, Cheng et al. [18] used a dimethylbipyridyl-copper (II) complex, as a metal cofactor interacting with the human telomeric G4 (HT21) [d(GGGTTAGGGTTAGGGTTAGGG)] (Figure 1) to build a DNA hybrid catalyst for enantioselective sulfoxidation of thioanisole, providing an ee of 56%.

A further study on the same reaction clarified the binding mode of the copper–bipyridine cofactor, the substrate, and HT21. It revealed non-specific interactions of the copper complexes and thioanisole with the second loop and the 3′ terminus of the G4 structure, confirming that the reaction generally occurs on a terminal G-quartet within a region encircled by the loop residues [19]. Indeed, it is proven that the catalytic performance of G4 DNA hybrid catalysts depends on the G4 structures [20] and that the loop sequence of the G4 DNA can influence the chiral expression of the reaction products [21]. Therefore, to expand the catalytic performance of HT21 as a DNA hybrid catalyst and to assess the role of its loop residues in modulating the enantioselectivity of thioanisole oxidation, we investigated a series of HT21 derivatives in which a single adenosine residue in the loops of this telomeric sequence was replaced with a modified analogue. These variants were examined in detail in our recent study [22].

Among these, the most remarkable performance was obtained by the HT21 analogue containing a β-L-2′-deoxyadenosine residue in the first loop (HT21-AL1), having been shown to provide an ee of 84%. A further contribution to understanding the mechanism of chiral control of sulfoxidation was provided by Spinelli et al. [23], corroborating that the reaction occurs at the 3′-tetrad and/or the second loop of the hybrid native HT21. These studies confirm a continued interest in further investigating the relationship between DNA G-quadruplexes and their enantioselective catalytic performance, the correlation between G4 topologies and ee in asymmetric synthesis, and the specific site on the G4 where the reaction occurs, as this may influence the formation of different enantiomers [23,24]. 

For these purposes, a series of HT21 derivatives has been synthesized, each sequence containing a single chemically modified thymidine replacing the natural one in the TTA loops (Table 1).

**Figure 2:**
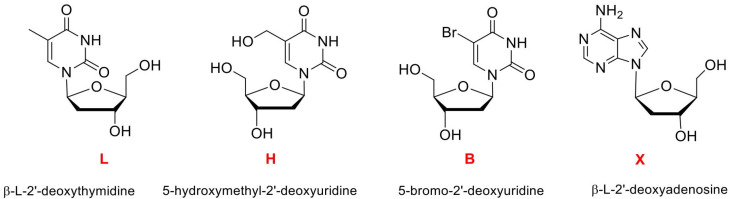
Modified monomers incorporated into HT21.

Chemical modifications were first included in a single thymidine loop residue near the upper or lower G-tetrad and introduced into the HT21 sequence using commercially available monomers, namely β-L-2′-deoxythymidine (**L**), 5-hydroxymethyl-2′-deoxyuridine (**H**), and 5-bromo-2-deoxyuridine (**B**) (Figure 2).

In this paper, we evaluate the effects of these straightforward chemical modifications on the catalytic activity of the HT21 hybrid catalyst in the enantioselective sulfoxidation of thioanisole. Subsequently, the catalytic properties of a second series of HT21 derivatives (Table 2) were tested under the same experimental conditions. These additional HT21 analogues were designed to further investigate the effect of the chemical modification in the first series of HT21 derivatives that produced on average the best results in all first thymidine loop positions (Table 1), now extended to the second thymidine (T) of the TTA loops.

Finally, to evaluate a possible synergistic effect between the more promising L modifications and the one that previously afforded the highest ee in our sulfoxidation study [22], we tested two additional doubly modified HT21 derivatives obtained by including β-L-2′-deoxythymidine (**L**) together with a modified adenosine residue (β-L-2′-deoxyadenosine, **X**) (Figure 2 and Table 2).

## 2. Results and Discussion

### 2.1. CD Spectroscopy

**Figure 3:**
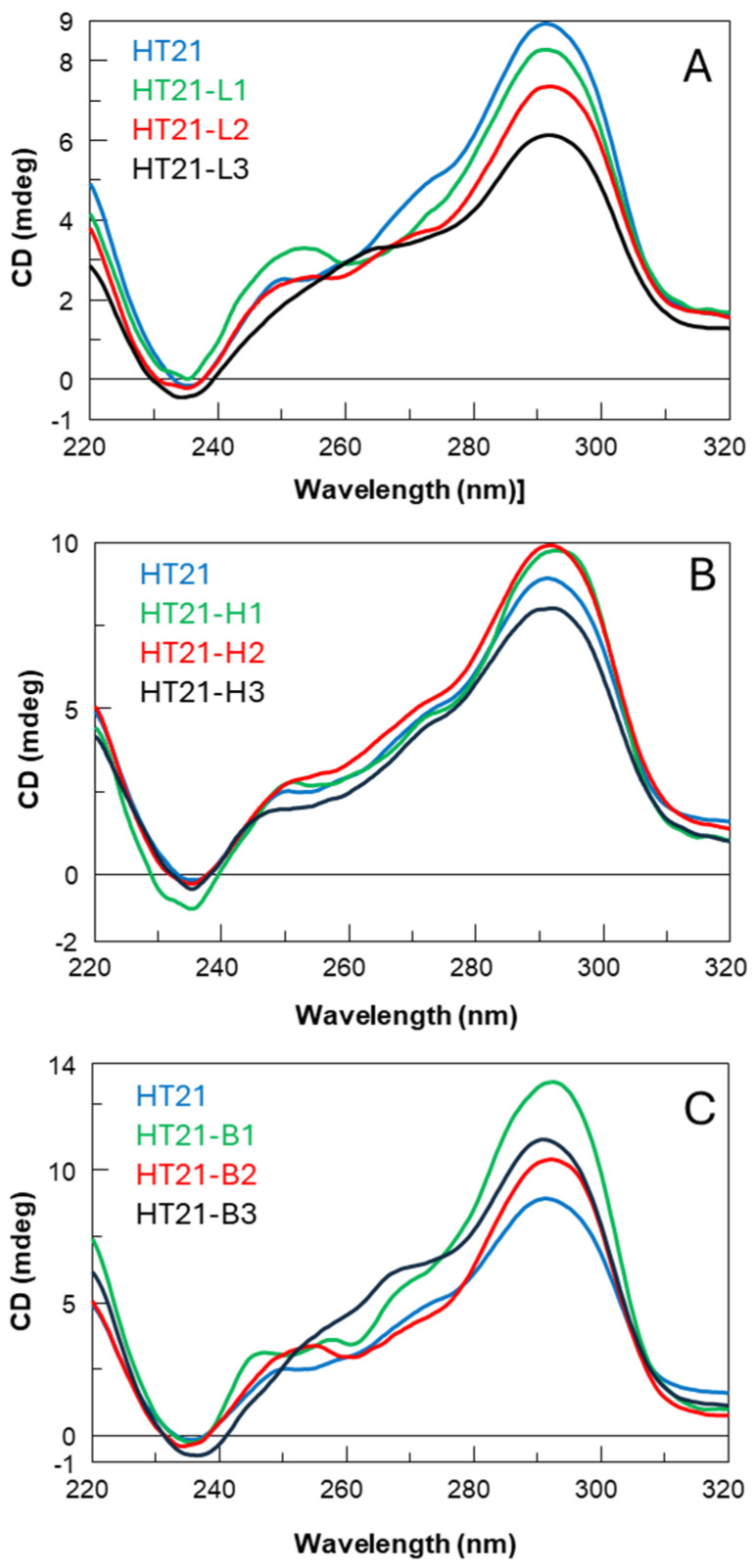
CD spectra of the first series of HT21 investigated derivatives (see Table 1; panel (**A**): **L** analogues; panel (**B**): **H** analogues; panel (**C**): **B** analogues) compared with the natural counterpart in 20 mM MOPS buffer (pH 7.0) containing 150 mM KCl (20 μM in ODN).

**Figure 4:**
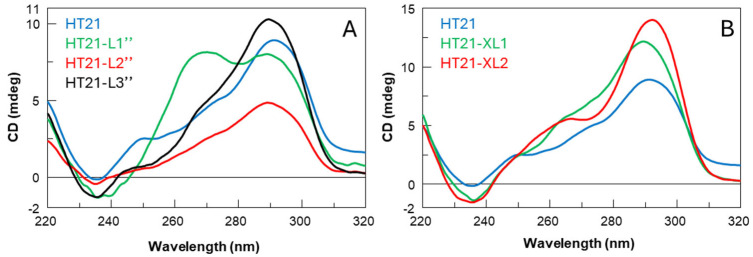
CD spectra of the second series of HT21 investigated derivatives (see Table 2; panel (**A**): **L″** analogues, panel (**B**): **XL** analogues) compared with the natural counterpart in 20 mM MOPS buffer (pH 7.0) containing 150 mM KCl (20 μM in ODN).

Circular Dichroic (CD) spectroscopy is one of the key tools for studies of the conformational properties of G4 structures [25] due to its high sensitivity and simplicity of preparation of samples to be analyzed. This technique can be used to validate the formation by nucleic acid sequences of specific structural arrangements, i.e., i-motifs, G-quadruplexes [26], and within the G4s CD can indicate the adoption of a particular folding topology. G4 topologies are grouped into parallel, antiparallel, or hybrid, and each of these shows a particular CD spectrum. It is known, in fact, that there is a close correlation between CD profiles and G4 topologies [27,28]. Therefore, comparing the CD profile of HT21 with those of its studied derivatives may allow us to identify closely related topological characteristics. After appropriate sample preparation and annealing processes in the reaction buffer (see below), CD spectra were measured for each analogue in comparison with the unmodified HT21 at the same temperature at which the oxidation reaction takes place (15 °C). In these experimental conditions, HT21 revealed the typical CD profile of hybrid-type telomeric G4s [29], characterized by a strong positive peak around 290 nm with a shoulder peak around 270 nm and a smaller negative peak at 240 nm. Concerning the first series of HT21 analogues (Figure 3A–C), all derivatives showed CD profiles almost superimposable onto each other and closely comparable to that of the natural one, particularly the H series (Figure 3B), apart from slight differences in band intensities and minimal band shifts in the region between 270 and 250 nm. These data confirm that the main conformation adopted by these HT21 analogues is the hybrid-type intramolecular G4 formed by the unmodified telomeric sequence, beyond slight local structural differences probably involving the loop containing the modified residues. More significant differences compared to the natural counterpart can be found in the CD spectra of the second series of derivatives (Figure 4A,B), especially for the HT21-L″ analogues. In particular, the CD spectrum of HT21-L1″, in addition to the maximum peak at 295 nm typical of hybrid G4s, showed a positive peak at around 264 nm of equal intensity, typical of parallel G4s [30], thus indicating the presence in solution of a complex mixture of G4s. Instead HT21-L2″ displayed a CD profile that follows the natural sequence trend but differs significantly from it in terms of intensity, revealing the presence in solution of a minor quantity of structured species. Only HT21-L3″ was demonstrated to preserve a CD profile very similar to that of the natural one (Figure 4A).

Concerning the HT21-XL derivatives, the substitution of two different residues in the same sequence contributes favorably to the formation in solution of folded species that maintain the HT21 hybrid G4 conformation, since both analogues showed CD profiles closely comparable to the natural one, except for the greater intensity of the bands (Figure 4B).

CD spectroscopy can also be used to evaluate the thermal stability of G4s by measuring the CD values at the maximum positive peak as a function of the temperature. 

Therefore, to determine the influence of loop unnatural monomers on the thermal stability of the HT21 G4 DNA catalyst, all investigated modified ODNs were exposed to CD denaturation experiments, and the obtained melting temperatures (T_m_) were compared with those of the parent sequence (Table 1 and Table 2). CD melting curves were registered in the same buffer of the oxidation reaction at 20 µM ODN concentration, by monitoring for each sample the CD values at the maximum cotton effect from 20 °C to 95 °C, subsequently normalized for better comparison (Figure S1). The HT21 analogues of each series showed highly similar CD melting profiles to each other, revealing T_m_ values ranging from a minimum of 69 °C to a maximum of 74 °C, thus equal to or slightly higher than the natural one (69 °C). These data indicate that both the substitution of a single thymidine and the simultaneous replacement of an adenosine and a thymidine in the loops of HT21 with the analyzed analogues contribute favorably to the thermal stability of the HT21 G4 DNA catalyst.

### 2.2. Investigation of the Catalytic Activity of HT21 Analogues 

**Scheme 1:**
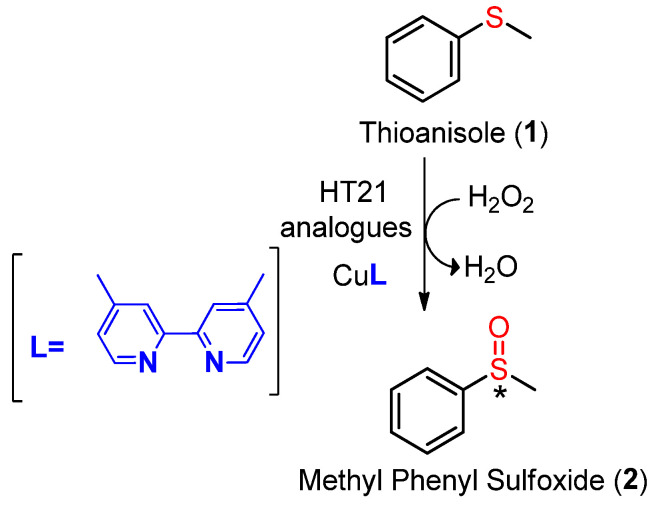
Enantioselective sulfoxidation of thioanisole (**1**) to yield methyl phenyl sulfoxide (**2**). The asterisk (*) indicates a stereogenic (chiral) center.

After the CD analysis, the catalytic properties of all investigated HT21 derivatives were evaluated in the enantioselective sulfoxidation of thioanisole (**1**), using HT21 analogues in combination with the Cu(II) complex of 4,4′-dimethyl-2,2′-bipyridine (CuL) and H_2_O_2_ as an oxidant (Scheme 1). All reactions were performed under the previously described and optimized conditions [18,22]. Control experiments were performed in the absence of HT21 and in the absence of both HT21 and the Cu**L** complex. Under the first conditions, the Cu–ligand complex alone afforded a small racemic mixture of sulfoxides (Figure S22), whereas in the second case no oxidation of thioanisole was observed at all (Figure S23). 

**Figure 5:**
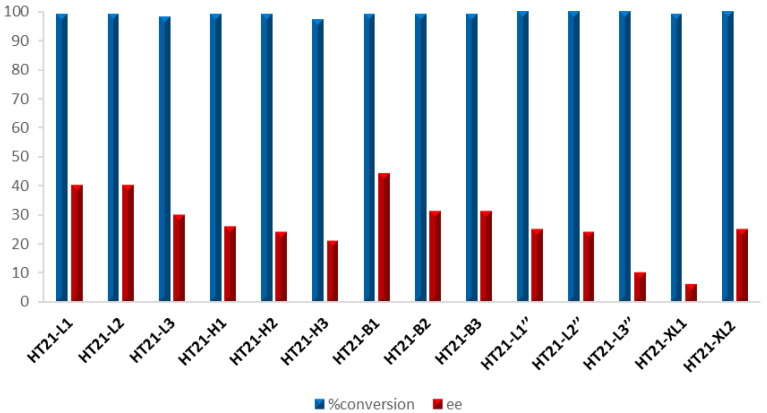
Enantioselective sulfoxidation of thioanisole (**1**) to methyl phenyl sulfoxide (**2**) catalyzed by HT21 analogues. % Conversion and % ee were determined by chiral-phase HPLC within reproducibility of 2%. The overoxidized product sulfone is untraceable.

Initially, chemical modifications were introduced at a single thymidine loop residue using the commercially available monomers β-L-2′-deoxythymidine (**L**), 5-hydroxymethyl-2′-deoxyuridine (**H**), and 5-bromo-2′-deoxyuridine (**B**). These modified sequences were designed to explore how alterations in the loop region, such as modification of the C-5 substitution of the pyrimidine nucleus or the incorporation of an L-sugar, could impact on both the catalytic efficiency and the enantioselectivity of the G4 DNA metalloenzyme in sulfoxidation reactions, probably by affecting the surrounding catalytic microenvironment and substrate orientation. Among these variants, the best results on average in all loops, and particularly in the second loop, where the reaction preferentially occurs in the case of hybrid native HT21 [19,23], were obtained with **L** derivatives, reaching an ee of 40% (Table 1, Figure 5).

As a next step, we also evaluated the effect of this modification on the second thymidine residue of the loops in the HT21 sequence, yielding the oligonucleotides HT21-L1″, HT21-L2″, and HT21-L3″. However, this modification likewise resulted in poor enantioselectivity, with an ee ranging from 10 to 25% (Table 2, Figure 5).

Finally, to evaluate whether combining the most effective adenine modification (AL1), obtained in our previous investigation, with the more promising thymidine substitution, namely **L**, could produce synergistic effects, two doubly modified HT21 derivatives, HT21-XL1 and HT21-XL2, were prepared and tested. The introduction of multiple loop substitutions in HT21-XL1 and HT21-XL2, as well as modification of the second T in the HT21 loops, resulted in a noticeable decrease in the enantiomeric excess achieved, compared to a single AL1 modification (6 and 25% ee, respectively) (Table 2, Figure 5). However, all investigated derivatives showed excellent catalytic properties in sulfoxidation, since all reactions proceeded with complete conversion of thioanisole as indicated in Figure 5. 

In addition, enantioselective sulfoxidation was investigated using benzyl methyl sulfide (**3**), a substrate that has not previously been employed in enantioselective oxidation reactions catalyzed by HT21 or its analogues. For this study, the most promising HT21 derivatives, namely HT21-AL1 and HT21-AL2, reported in our previous work [22], together with HT21-L1, HT21-L2, and HT21-B1 described in the present study, were selected. When this alternative substrate was used, a marked change in enantioselective behavior was observed. Both native HT21 and its analogues led to complete conversion of the sulfide to the corresponding sulfoxide; however, in all cases poor enantioselectivities were observed (Table S1).

Overall, these results highlight the crucial role of the loop regions in proximity to the terminal G-tetrads in governing the chiral induction during DNA-based catalysis. These data expand our understanding of G4 DNA catalytic properties in asymmetric oxidations, while indicating that additional modifications were detrimental to the overall reaction performance. 

## 3. Materials and Methods

### 3.1. Oligonucleotide Synthesis and Purification

The oligonucleotides reported in Table 1 and Table 2 were synthesized on a K&A H-16 DNA synthesizer (K&A Labs GmBH, Schaafheim, Germany) using solid-phase β-cyanoethyl phosphoramidite chemistry at a 5 µmol scale. The modified monomers were introduced in the sequences using commercially available 5-bromo-2′-deoxyuridine, 3′-[(2-cyanoethyl)-N,N-diisopropyl]-phosphoramidite, 5-acetoxymethyl-2′-deoxyUridine,3′-[(2-cyanoethyl)-(N,N-diisopropyl)]-phosphoramidite (LGC Biosearch Technologies, Guildford, UK), β-L-2′-deoxyThymidine,3′-[(2-cyanoethyl)-(N,N-diisopropyl)]-phosphoramidite, and β-L-N6-benzoyldeoxyAdenosine-3′-[(2-cyanoethyl)-(N,N-diisopropyl)]-phosphoramidite (ChemGenes Corporation, Wilmington, MA, USA). The oligomers were detached from the solid support and deprotected by treatment with concentrated aqueous ammonia at room temperature for 24 h (HT21-B derivatives) or at 55 °C overnight (all others). The combined filtrates and washings were concentrated under reduced pressure, redissolved in H_2_O, analyzed, and purified by high-performance liquid chromatography on a Nucleogel SAX column (Macherey-Nagel, 1000-8/46, Düren, Germany), using buffer A, 20 mM NaH_2_PO_4_/Na_2_HPO_4_ aqueous solution (pH 7.0) containing 20% (*v*/*v*) CH_3_CN, and buffer B, 1 M NaCl, 20 mM NaH_2_PO_4_/Na_2_HPO_4_ aqueous solution (pH 7.0) containing 20% (*v*/*v*) CH_3_CN; a linear gradient from 0 to 100% B for 60 min and a flow rate 1 mL/min were used. The collected fractions of the oligomers were desalted by Sep-pak cartridges (C-18).

### 3.2. Chemicals

The ligand **L** (4,4′-Dimethyl-2,2′-bipyridine) and thioanisole were purchased from Fluorochem. Hydrogen peroxide solution (3%), Cu(NO_3_)_2_•3H_2_O, and 3-(N-morpholino)propanesulfonic acid (MOPS) were obtained from Sigma-Aldrich (Merck, Darmstadt, Germany). 

The complex Cu**L** was synthesized as previously described [31] and dissolved in water. In detail, **L** (100 mg, 0.54 mmol) and Cu(NO_3_)_2_•3H_2_O (132 mg, 0.54 mmol) were dissolved in ethanol. The solution was stirred for 1 h at room temperature until a blue solid precipitate formed. The precipitate was filtered, washed with ethanol and diethyl ether, and then dried in an oven (yield: 95%; ESI-MS: *m*/*z* 309.1 [L–Cu^2+^–NO_3_^−^]^+^).

### 3.3. CD Measurements 

CD samples of HT21 and its derivatives listed in Table 1 and Table 2 were prepared at an oligonucleotide concentration of 20 µM using 20 mM MOPS buffer (pH 7.0) containing 150 mM KCl. After dilution in the buffer, each sample was submitted to the annealing procedure by heating to 90 °C and slowly cooling to room temperature. CD spectra and CD melting profiles of all G-quadruplexes were recorded on a Jasco 715 CD spectrophotometer (JASCO Corporation, Hachioji, Tokyo, Japan). For the CD spectra, the wavelength was varied from 320 to 220 nm with a scan rate of 100 nm min^−1^, a response of 16 s, and a bandwidth of 2.0 nm. The spectra presented in Figure 3 and Figure 4 were normalized by subtraction of the background scan with buffer. The temperature was kept constant at 15 °C with a thermoelectrically controlled cell holder (Jasco PTC-348, JASCO Corporation, Hachioji, Tokyo, Japan). CD melting curves were registered as a function of the temperature (range: 20–95 °C) for all G4 complexes at the wavelengths of their maximum Cotton effect. The CD data were recorded in a 0.1 cm pathlength cuvette with a scan rate of 0.5 °C/min. Each measurement was the average of three scans.

### 3.4. Oxidation Procedure

All reactions of sulfoxidation were thermostatted at 15 °C and agitated by using a Thermomixer C (Eppendorf S.r.l., Milan, Italy).

G4 DNA catalyst (10 µM) was dissolved in MOPS buffer (1 mL, 20 mM, pH 7.0) containing KCl (150 mM), heated for 3 min at 98 °C, and then slowly cooled to room temperature over 2 h. The copper complex CuL in water (50 µM) was added and stirred for 30 min at 15 °C. Then, 10 µL of a 0.5 M solution of thioanisole in CH_3_CN was added, followed by H_2_O_2_ (7.5 µL of a 3% aqueous solution). The reaction mixture was stirred for 5 h at 15 °C, o.n. At the end of the reaction, diethyl ether (3 × 3 mL) was added to extract the products. Anhydrous Na_2_SO_4_ was added to the combined organic layers, and the solvent was removed under reduced pressure. The crude products were analyzed directly by HPLC using a chiral column. All products and substrates were quantified and characterized by using calibration curves of the corresponding commercially available compounds. Yields were evaluated using calibration curves. ee was calculated according to the following equation:

$$\frac{A_{2}\;-A_{1}}{A_{1}+A_{2}}$$

where A_1_ and A_2_ are the area under the chromatographic peak of the first (*R*) and second (*S*) eluted enantiomers, respectively. The determination of the *R* and *S* enantiomers was carried out based on previously reported literature data [13], and the enantiomeric excess is reported with respect to the predominant *S* enantiomer.

Purification was carried out using an Agilent 1260 Infinity II quaternary pump (Agilent Technologies, Santa Clara, CA, USA) connected to a 1260 Infinity II UV/Vis detector equipped with a 1260 Infinity II manual injector (Agilent Technologies, Santa Clara, CA, USA) and a Jasco PU-2089 Plus Quaternary Gradient Pump (JASCO Corporation, Hachioji, Tokyo, Japan) connected to a UV-2075 Plus UV/Vis equipped with a Waters Rheodyne injector (Waters Corporation—Milford, MA, USA). HPLC analysis was performed at a wavelength of 254 nm, using *n*-hexane and isopropanol (*i*-PrOH) (9:1) as an eluent and a Lux^®^ Cellulose-1 column (Phenomenex, 5 µm, 250 × 4.6 mm) (Phenomenex Inc., Torrance, CA, USA).

- Methyl Phenyl Sulfoxide (**2**): ^1^H NMR (CD_3_OD, 400 MHz): δ_H_ 7.71 (2H, dd, *J* = 7.8 and 2.0 Hz), 7.56 (3H, m), 2.77 (3H, s). ^13^C NMR (CD_3_OD, 100 MHz): δ_C_ 146.0, 132.3 (2C), 130.5 (2C), 124.7, 43.8. ESI-MS 140.9 [M+H]^+^
- Benzyl Methyl Sulfoxide (**4**): ^1^H NMR (CDCl_3_, 400 MHz): δ_H_ 7.40 (3H, m), 7.31 (2H, dd, *J* = 7.7 and 1.9 Hz), 4.14 (1H, d, *J* = 12.8 Hz), 3.98 (1H, d, *J* = 12.8 Hz), 2.50 (3H, s).

## 4. Conclusions

In summary, we found that specific thymidine loop modifications can affect the enantioselective catalytic performance of telomeric G4 DNAzymes in sulfoxidation reactions. A comprehensive examination of chemical substitutions systematically introduced in definite positions of TTA loops in the HT21 sequence allowed us to thoroughly evaluate the contributions of individual loop residues to the chiral induction process. Our results revealed that all loop regions of HT21 can be involved in modulating enantioselectivity, contributing to creation of the appropriate stereochemical environment. Particularly, whereas the most favorable chemical modification remains the β-L-2′-deoxyadenosine introduction in the first loop (HT21-AL1), inducing an enantiomeric excess of 84%, thymidine substitution, either singly or in combination, produced an ee decrease despite reserving a complete substrate conversion. The systematic comparison of different loop modifications suggests that although T-modified substitutions can affect the G4 chiral environment, positions within the same loop are not equivalent and single substitutions within the same loop can result in different enantioselectivities.

These data imply that the steric and electronic properties of all G4 loops can influence the catalytic performance of the DNAzyme, although several studies demonstrated that the reaction occurs at the 3′-tetrad and/or the second loop in the case of hybrid native HT21 [19,23]. We hypothesize that the introduction of T residues, modified at the sugar configuration or at the C-5 position of the base, in the loop sequences could disturb the correct orientation of the substrate in the catalytic site or influence the interaction with the Cu (II) cofactor, thus failing the enantiomeric selection. This effect seems to be more evident when modifications involve the third loop. Additionally, the diminished enantioselectivity obtained by doubly modified HT21 derivatives (HT21-XL1 and HT21-XL2) not only reveals the absence of synergistic effects, but also suggests that specific combinations could establish detrimental steric or electronic effects with the catalytic site as well. Looking ahead, future research could focus on exploring other types of substrates and/or metal cofactors to evaluate the general applicability of modified G4 catalysts in other asymmetric oxidation reactions and/or joining G4 catalysts into hybrid systems with other catalytic domains or cofactors to improve selectivity and functional versatility. Structural characterization techniques (e.g., CD spectroscopy, NMR, molecular modeling) combined with catalytic assays could help us to better understand the correlation between conformational changes and reaction outcomes. Finally, this work contributes to the growing body of knowledge on the rational design of DNA-based catalysts with improved performance and broader applicability in enantioselective synthesis, providing useful insights on how individual loop residues contribute to asymmetric induction and offering further details for tuning G4-based catalytic scaffolds for pharmaceutical and synthetic applications.

## Figures and Tables

**Table 1:** Sequences of the first series of HT21 derivatives investigated, melting temperatures (T_m_) of the adopted G4 and enantiomeric excess (ee) percentages. **L**: β-L-2′-deoxythymidine; **H**: 5-hydroxymethyl-2′-deoxyuridine; **B**: 5-bromo-2-deoxyuridine. The modified monomers are highlighted in red. * As reported by Festa et al. [22]. ^a^ Determined by chiral-phase HPLC within reproducibility of 2%. The enantiomeric excess is reported with respect to the predominant *S* enantiomer. HPLC chromatograms are reported in Electronic Supplementary Materials.

Name	Sequence (5′-3′)	T_m_ °C (±1)	ee % ^a^
HT21	GGGTTAGGGTTAGGGTTAGGG	69 *	56 *
HT21-L1	GGG**L**TAGGGTTAGGGTTAGGG	71	40
HT21-L2	GGGTTAGGG**L**TAGGGTTAGGG	73	40
HT21-L3	GGGTTAGGGTTAGGG**L**TAGGG	74	30
HT21-H1	GGG**H**TAGGGTTAGGGTTAGGG	69	26
HT21-H2	GGGTTAGGG**H**TAGGGTTAGGG	72	24
HT21-H3	GGGTTAGGGTTAGGG**H**TAGGG	71	21
HT21-B1	GGG**B**TAGGGTTAGGGTTAGGG	73	43
HT21-B2	GGGTTAGGG**B**TAGGGTTAGGG	73	31
HT21-B3	GGGTTAGGGTTAGGG**B**TAGGG	73	30

**Table 2:** Sequences of the second series of HT21 derivatives investigated, melting temperatures (T_m_) of the adopted G4, and enantiomeric excess (ee) percentages. **L**: β-L-2′-deoxythymidine; **X**: β-L-2′-deoxyadenosine. The modified monomers are highlighted in red. ^a^ Determined by chiral-phase HPLC within reproducibility of 2%. The enantiomeric excess is reported with respect to the predominant *S* enantiomer. HPLC chromatograms are reported in Electronic Supplementary Materials.

Name	Sequence (5′-3′)	T_m_ °C (±1)	ee % ^a^
HT21-L1″	GGGT**L**AGGGTTAGGGTTAGGG	72	25
HT21-L2″	GGGTTAGGGT**L**AGGGTTAGGG	71	24
HT21-L3″	GGGTTAGGGTTAGGGT**L**AGGG	71	10
HT21-XL1	GGG**L**T**X**GGGTTAGGGTTAGGG	74	6
HT21-XL2	GGGTT**X**GGG**L**TAGGGTTAGGG	74	25

## Data Availability

The data presented in this study are available on request from the corresponding authors.

## Supplementary Materials

The following supporting information can be downloaded at https://www.mdpi.com/article/10.3390/molecules31030442/s1, Table S1: Enantioselective sulfoxidation of benzyl methyl sulfide; Figure S1: CD melting profiles; Figure S2: HPLC chromatogram of sulfoxidation of thioanisole (**1**) catalyzed by HT21-L1; Figure S3: HPLC chromatogram of sulfoxidation of thioanisole (**1**) catalyzed by HT21-L2; Figure S4: HPLC chromatogram of sulfoxidation of thioanisole (**1**) catalyzed by HT21-L3; Figure S5: HPLC chromatogram of sulfoxidation of thioanisole (**1**) catalyzed by HT21-H1; Figure S6: HPLC chromatogram of sulfoxidation of thioanisole (**1**) catalyzed by HT21-H2; Figure S7: HPLC chromatogram of sulfoxidation of thioanisole (**1**) catalyzed by HT21-H3; Figure S8: HPLC chromatogram of sulfoxidation of thioanisole (**1**) catalyzed by HT21-B1; Figure S9: HPLC chromatogram of sulfoxidation of thioanisole (**1**) catalyzed by HT21-B2; Figure S10: HPLC chromatogram of sulfoxidation of thioanisole (**1**) catalyzed by HT21-B3; Figure S11: HPLC chromatogram of sulfoxidation of thioanisole (**1**) catalyzed by HT21-L1″; Figure S12: HPLC chromatogram of sulfoxidation of thioanisole (**1**) catalyzed by HT21-L2″; Figure S13: HPLC chromatogram of sulfoxidation of thioanisole (**1**) catalyzed by HT21- L3″; Figure S14: HPLC chromatogram of sulfoxidation of thioanisole (**1**) catalyzed by HT21-XL1; Figure S15: HPLC chromatogram of sulfoxidation of thioanisole (**1**) catalyzed by HT21-XL2; Figure S16: HPLC chromatogram of sulfoxidation of benzyl methyl sulfide (**3**) catalyzed by HT21; Figure S17: HPLC chromatogram of sulfoxidation of benzyl methyl sulfide (**3**) catalyzed by HT21-AL1; Figure S18: HPLC chromatogram of sulfoxidation of benzyl methyl sulfide (**3**) catalyzed by HT21-AL2; Figure S19: HPLC chromatogram of sulfoxidation of benzyl methyl sulfide (**3**) catalyzed by HT21-L1; Figure S20: HPLC chromatogram of sulfoxidation of benzyl methyl sulfide (**3**) catalyzed by HT21-L2; Figure S21: HPLC chromatogram of sulfoxidation of benzyl methyl sulfide (**3**) catalyzed by HT21-B1; Figure S22: HPLC chromatogram of sulfoxidation of thioanisole (**1**) without HT21; Figure S23: HPLC chromatogram of sulfoxidation of thioanisole (**1**) without HT21 and CuL; Figure S24: ^1^H NMR spectrum of methyl phenyl sulfoxide (**2**) CDCl_3_, 400 MHz); Figure S25: ^13^C NMR spectrum of methyl phenyl sulfoxide (**2**) (CDCl3, 100 MHz); Figure S26: ESI-MS spectrum of methyl phenyl sulfoxide (**2**) [M+H]^+^; Figure S27. ^1^H NMR spectrum of benzyl methyl sulfoxide (**4**) (CDCl3, 400 MHz).

## Abbreviations

The following abbreviations are used in this manuscript:

ee	Enantiomeric excess
CD	Circular dichroism
G4	G-quadruplex
