# Supplementary material for: Impact of Thymidine Loop Modifications on Telomeric G-Quadruplex Catalytic Systems for Asymmetric Sulfoxidation
Source: Molecules. 2026 Jan 27;31(3):442. doi: 10.3390/molecules31030442 (PMC12898609; doi:10.3390/molecules31030442)

Supplementary Material

Impact of Thymidine Loop Modifications on Telomeric G-Quadruplex Catalytic Systems for Asymmetric Sulfoxidation

Claudia Finamore †, Carmen Festa †, Daniela Benigno, Carla Aliberti, Rosa Barbato, Simona De Marino, Aldo Galeone, Veronica Esposito * and Antonella Virgilio *

Department of Pharmacy, University of Naples Federico II, Via D. Montesano 49, I-80131 Naples, Italy;
claudia.finamore@unina.it (C.F.); carmen.festa@unina.it (C.F.); daniela.benigno@unina.it (D.B.); carla.aliberti@unina.it (C.A.); rosa.barbato3@unina.it (R.B.); sidemari@unina.it (S.D.M.); galeone@unina.it (A.G.)
* Correspondence: verespos@unina.it (V.E.); antonella.virgilio@unina.it (A.V.)
† These authors contributed equally to this work.

Table of contents

Figure S1. CD melting profiles……………………………………………………………………………….3
Table S1. Enantioselective sulfoxidation of benzyl methyl sulfide (**3**)…………………………..……..4
Figure S2. HPLC chromatogram of sulfoxidation of thioanisole (**1**) catalyzed by HT21-L1……….……5
Figure S3. HPLC chromatogram of sulfoxidation of thioanisole (**1**) catalyzed by HT21-L2…………......5
Figure S4. HPLC chromatogram of sulfoxidation of thioanisole (**1**) catalyzed by HT21-L3…..……...…5
Figure S5. HPLC chromatogram of sulfoxidation of thioanisole (**1**) catalyzed by HT21-H1…..……........6
Figure S6. HPLC chromatogram of sulfoxidation of thioanisole (**1**) catalyzed by HT21-H2…..................6
Figure S7. HPLC chromatogram of sulfoxidation of thioanisole (**1**) catalyzed by HT21-H3…………....6
Figure S8. HPLC chromatogram of sulfoxidation of thioanisole (**1**) catalyzed by HT21-B1…………..…7
Figure S9. HPLC chromatogram of sulfoxidation of thioanisole (**1**) catalyzed by HT21- B2…………....7
Figure S10. HPLC chromatogram of sulfoxidation of thioanisole (**1**) catalyzed by HT21-B3…………....7
Figure S11. HPLC chromatogram of sulfoxidation of thioanisole (**1**) catalyzed by HT21-L1′′…………..8
Figure S12. HPLC chromatogram of sulfoxidation of thioanisole (**1**) catalyzed by HT21-L2′′………..…8
Figure S13. HPLC chromatogram of sulfoxidation of thioanisole (**1**) catalyzed by HT21- L3′′…….….....8
Figure S14. HPLC chromatogram of sulfoxidation of thioanisole (**1**) catalyzed by HT21-XL1…………9
Figure S15. HPLC chromatogram of sulfoxidation of thioanisole (**1**) catalyzed by HT21-XL2……….....9
Figure S16. HPLC chromatogram of sulfoxidation of benzyl methyl sulfide (**3**) catalyzed by HT21.….10
Figure S17. HPLC chromatogram of sulfoxidation of benzyl methyl sulfide (**3**) catalyzed by HT21-AL1……………………………………………………………………………………………………10
Figure S18. HPLC chromatogram of sulfoxidation of benzyl methyl sulfide (**3**) catalyzed by HT21-AL2……………………………………………………………………………………………………11
Figure S19. HPLC chromatogram of sulfoxidation of benzyl methyl sulfide (**3**) catalyzed by HT21-L1…...11
Figure S20. HPLC chromatogram of sulfoxidation of benzyl methyl sulfide (**3**) catalyzed by HT21-L2…..12
Figure S21. HPLC chromatogram of sulfoxidation of benzyl methyl sulfide (**3**) catalyzed by HT21-B1……………………………………………………………………………………………….....12
Figure S22. HPLC chromatogram of sulfoxidation of thioanisole (**1**) with Cu**L** and without HT21……………………………………………………………………………………………..…13
Figure S23. HPLC chromatogram of sulfoxidation of thioanisole (**1**) without HT21 and Cu**L**…………13

Figure S24. ^{1}H NMR spectrum of methyl phenyl sulfoxide (**2**) CD_3OD, 400 MHz)……………………………………………………………………………………………..…14
Figure S25. ^{13}C NMR spectrum of methyl phenyl sulfoxide (**2**) (CD_3OD, 100 MHz)……………………………………………………………………………………………….14
Figure S26. ESI-MS spectrum of methyl phenyl sulfoxide (**2**) $[M+H]^+$……………………………......15
Figure S27. ^{1}H NMR spectrum of benzyl methyl sulfoxide (**4**) ($CDCl_3$, 400 MHz)…………………….......15

Figure S1. Normalized CD melting profiles of analysed ODNs registered as a function of temperature for all modified quadruplexes at their maximum Cotton effect wavelengths. CD data were recorded in a 0.1 cm pathlength cuvette with a scan rate of 30°C/h at 20 μM ODN strand concentration in 20 mM MOPS buffer (pH 7.0) containing 150 mM KCl.

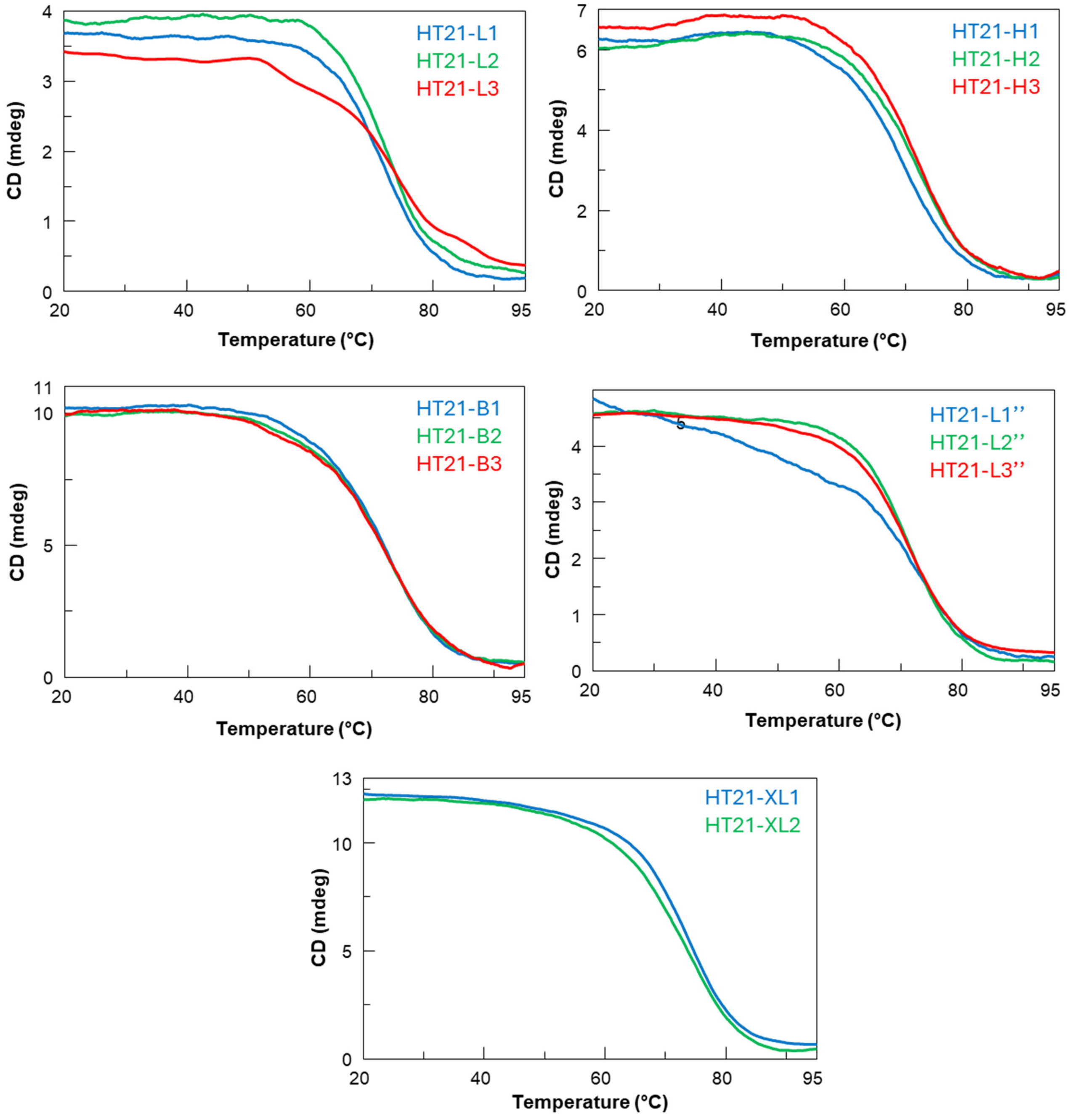

Table S1. Enantioselective sulfoxidation of benzyl methyl sulfide (**3**) to benzyl methyl sulfoxide (**4**) catalyzed by HT21 and its analogues. % Conversion and % ee were determined by chiral-phase HPLC within reproducibility of 2%.

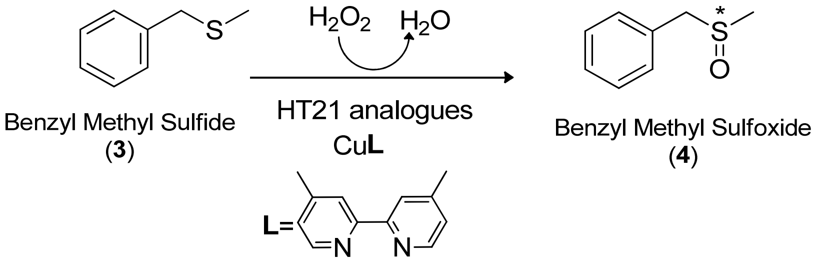

G4-DNA	%Conversion	ee%
HT21	100	<5
HT21-AL1 [Ref 22]	100	<5
HT21-AL2 [Ref 22]	100	8
HT21-L1	100	7
HT21-L2	100	<5
HT21-B1	100	5

Figure S2. HPLC chromatogram of sulfoxidation of thioanisole (**1**) catalyzed by HT21-L1.

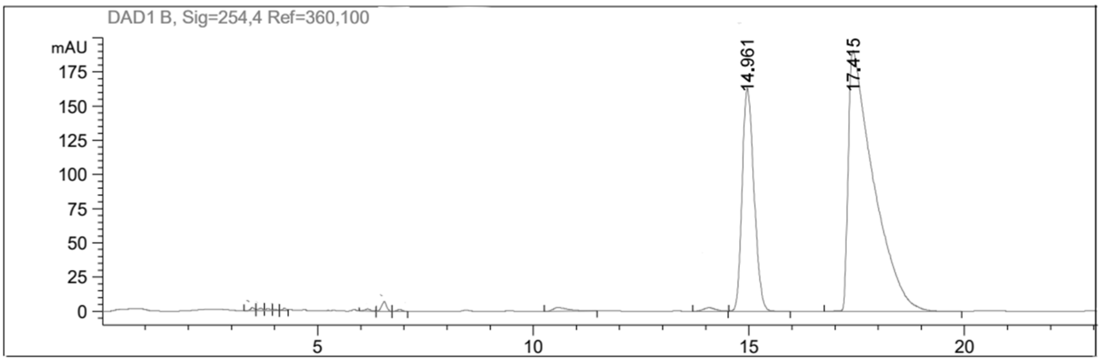

Peak Name	Rt (min)	Area	Factor
Thioanisole	4.226	10.86002	1.000
(R)-methylphenyl sulfoxide	14.961	3104.95898	1.000
(S)-methylphenyl sulfoxide	17.415	7706.00244	1.000

Figure S3. HPLC chromatogram of sulfoxidation of thioanisole (**1**) catalyzed by HT21-L2.

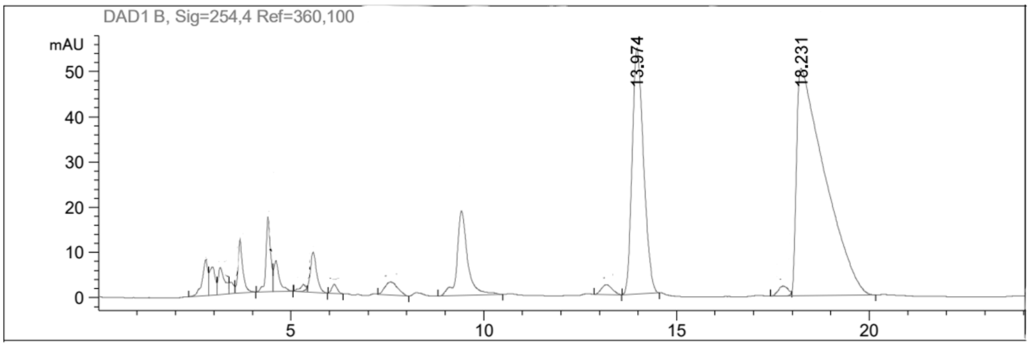

Peak Name	Rt (min)	Area	Factor
Thioanisole	4.401	140.70872	1.000
(R)-methylphenyl sulfoxide	13.974	1118.65210	1.000
(S)-methylphenyl sulfoxide	18.231	2558.03418	1.000

Figure S4. HPLC chromatogram of sulfoxidation of thioanisole (**1**) catalyzed by HT21-L3.

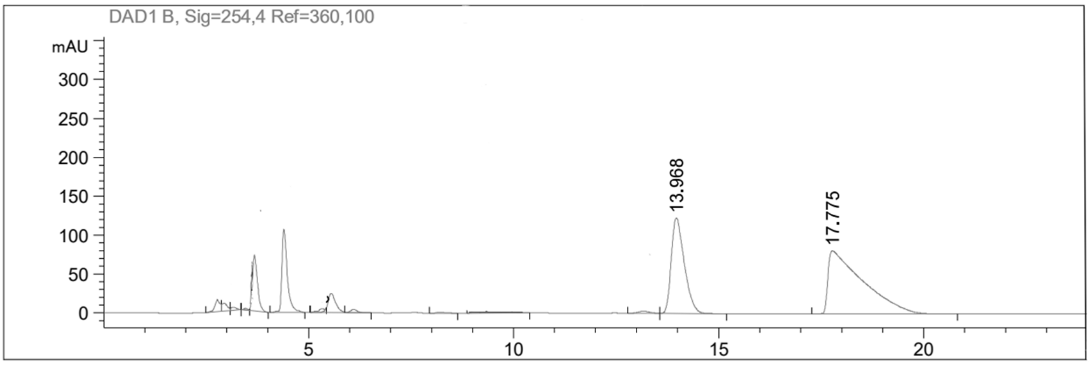

Peak Name	Rt (min)	Area	Factor
Thioanisole	4.400	974.52789	1.000
(R)-methylphenyl sulfoxide	13.968	2750.59497	1.000
(S)-methylphenyl sulfoxide	17.775	4800.47314	1.000

Figure S5. HPLC chromatogram of sulfoxidation of thioanisole (**1**) catalyzed by HT21-H1.

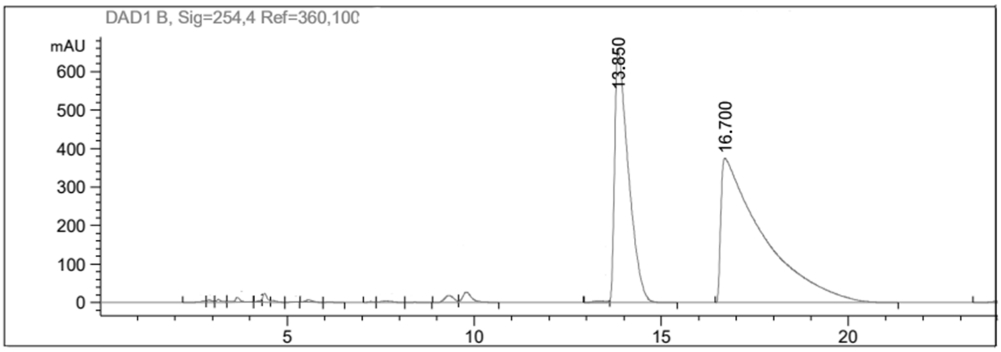

Peak Name	Rt (min)	Area	Factor
Thioanisole	4.394	196.03633	1.000
(R)-methylphenyl sulfoxide	13.850	1.67184e4	1.000
(S)-methylphenyl sulfoxide	16.700	2.93167e4	1.000

Figure S6. HPLC chromatogram of sulfoxidation of thioanisole (**1**) catalyzed by HT21-H2.

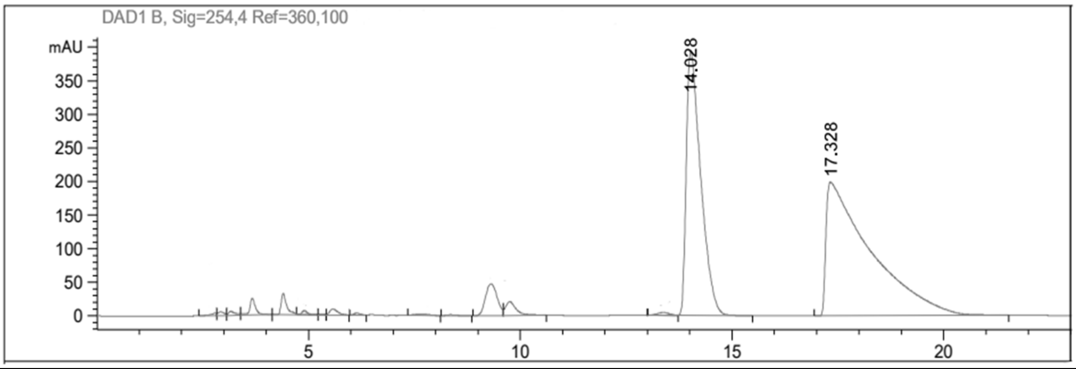

Peak Name	Rt (min)	Area	Factor
Thioanisole	4.403	301.94534	1.000
(R)-methylphenyl sulfoxide	14.028	9369.02441	1.000
(S)-methylphenyl sulfoxide	17.328	1.46274e4	1.000

Figure S7. HPLC chromatogram of sulfoxidation of thioanisole (**1**) catalyzed by HT21-H3.

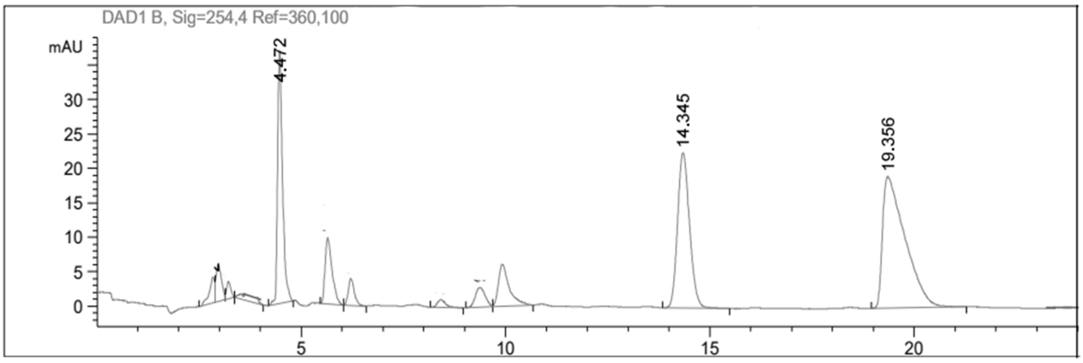

Peak Name	Rt (min)	Area	Factor
Thioanisole	4.472	307.29498	1.000
(R)-methylphenyl sulfoxide	14.345	460.33020	1.000
(S)-methylphenyl sulfoxide	19.356	732.15979	1.000

Figure S8. HPLC chromatogram of sulfoxidation of thioanisole (**1**) catalyzed by HT21-B1.

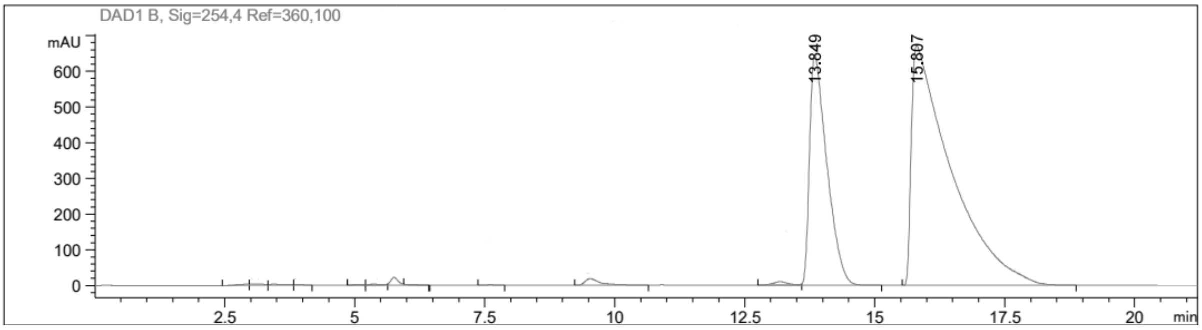

Peak Name	Rt (min)	Area	Factor
Thioanisole	5.035	19.46035	1.000
(R)-methylphenyl sulfoxide	13.849	1.495136e4	1.000
(S)-methylphenyl sulfoxide	15.807	3.55847e4	1.000

Figure S9. HPLC chromatogram of sulfoxidation of thioanisole (**1**) catalyzed by HT21- B2.

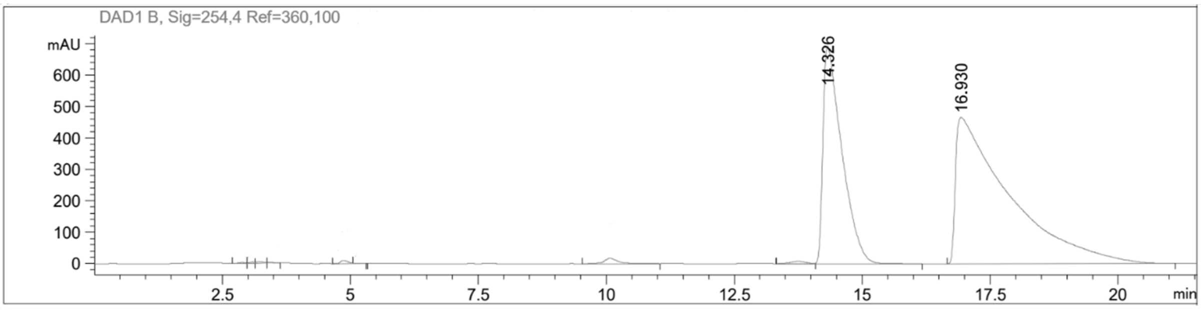

Peak Name	Rt (min)	Area	Factor
Thioanisole	4.863	105.26025	1.000
(R)-methylphenyl sulfoxide	14.326	1.76966e4	1.000
(S)-methylphenyl sulfoxide	16.930	3.38285e4	1.000

Figure S10. HPLC chromatogram of sulfoxidation of thioanisole (**1**) catalyzed by HT21-B3.

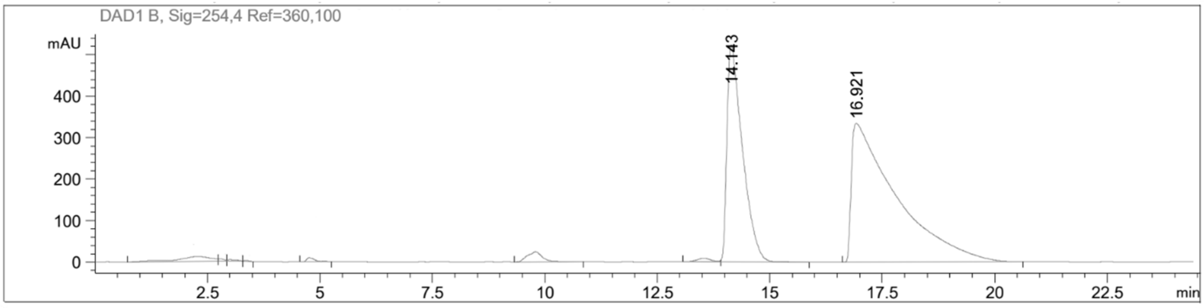

Peak Name	Rt (min)	Area	Factor
Thioanisole	4.768	116.73699	1.000
(R)-methylphenyl sulfoxide	14.143	1.24995e4	1.000
(S)-methylphenyl sulfoxide	16.921	2.30756e4	1.000

Figure S11. HPLC chromatogram of sulfoxidation of thioanisole (**1**) catalyzed by HT21-L1′′.

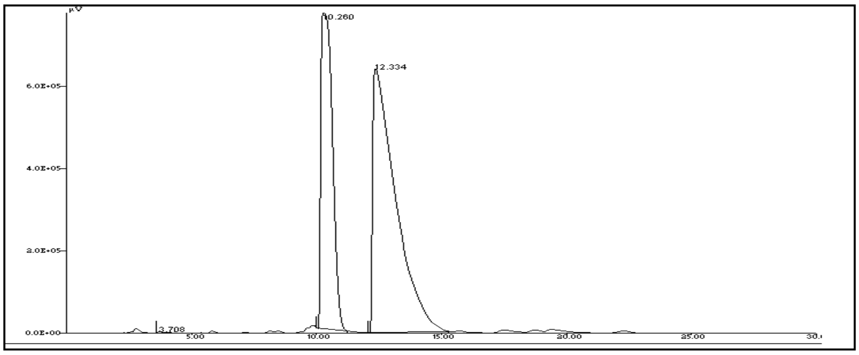

Peak Name	Rt (min)	Area	Factor
Thioanisole	3.708	21800,08	1.000
(R)-methylphenyl sulfoxide	10.260	22686576,81	1.000
(S)-methylphenyl sulfoxide	12.334	39215290,87	1.000

Figure S12. HPLC chromatogram of sulfoxidation of thioanisole (**1**) catalyzed by HT21-L2′′.

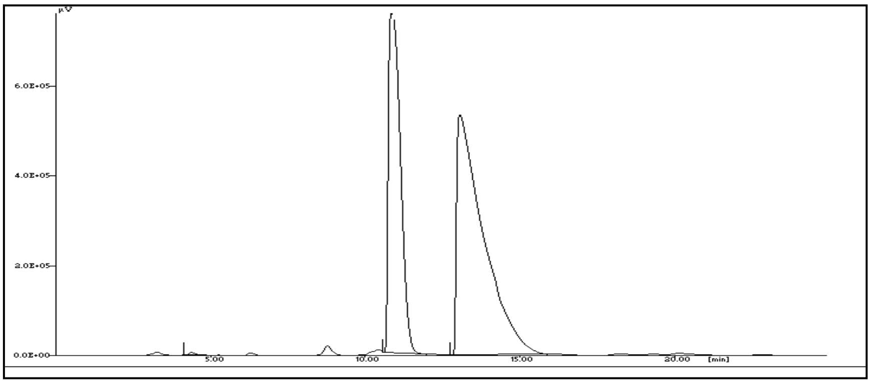

Peak Name	Rt (min)	Area	Factor
Thioanisole	4.372	56935,47	1.000
(R)-methylphenyl sulfoxide	10.827	18879704,15	1.000
(S)-methylphenyl sulfoxide	13.040	30946648,01	1.000

Figure S13. HPLC chromatogram of sulfoxidation of thioanisole (**1**) catalyzed by HT21- L3′′.

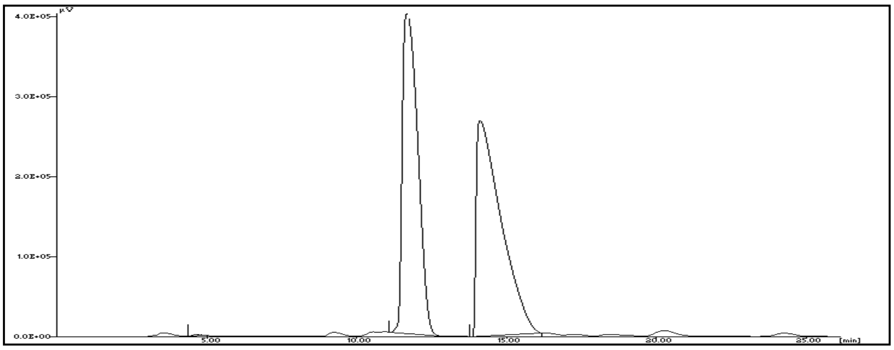

Peak Name	Rt (min)	Area	Factor
Thioanisole	4.655	35637,31	1.000
(R)-methylphenyl sulfoxide	11.651	13378043,99	1.000
(S)-methylphenyl sulfoxide	14.093	14820478,96	1.000

Figure S14. HPLC chromatogram of sulfoxidation of thioanisole (**1**) catalyzed by HT21-XL1.

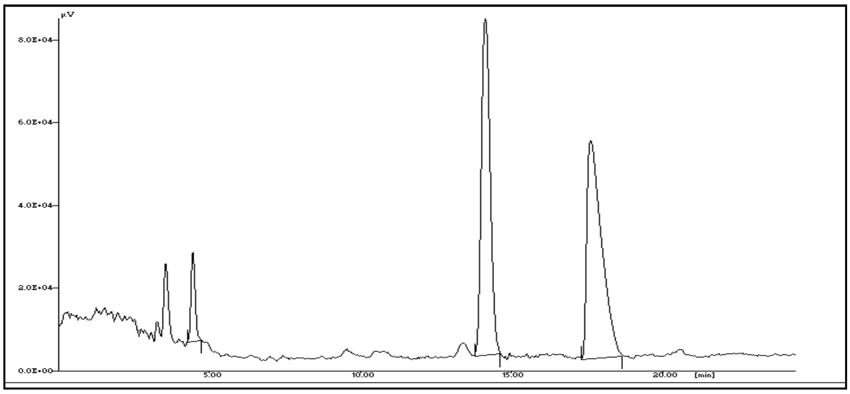

Peak Name	Rt (min)	Area	Factor
Thioanisole	4.427	160561,62	1.000
(R)-methylphenyl sulfoxide	14.114	1474030,01	1.000
(S)-methylphenyl sulfoxide	17.608	1666633,53	1.000

Figure S15. HPLC chromatogram of sulfoxidation of thioanisole (**1**) catalyzed by HT21-XL2.

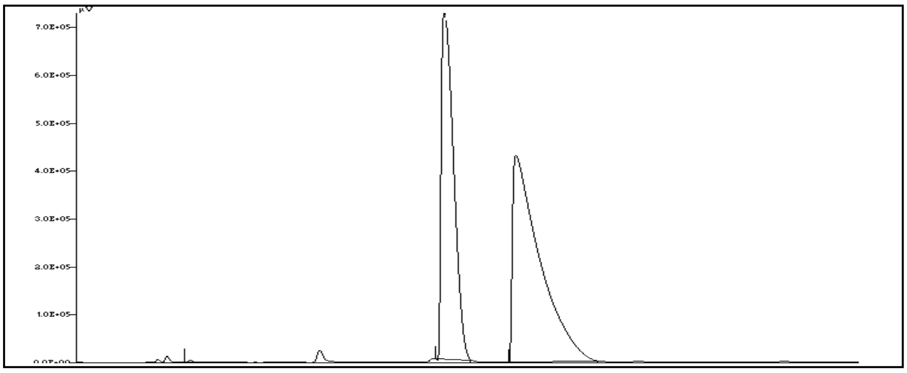

Peak Name	Rt (min)	Area	Factor
Thioanisole	4.352	23277,77	1.000
(R)-methylphenyl sulfoxide	14.073	21810702,64	1.000
(S)-methylphenyl sulfoxide	16.823	30088965,47	1.000

Figure S16. HPLC chromatogram of sulfoxidation of benzyl methyl sulfide (**3**) catalyzed by HT21.

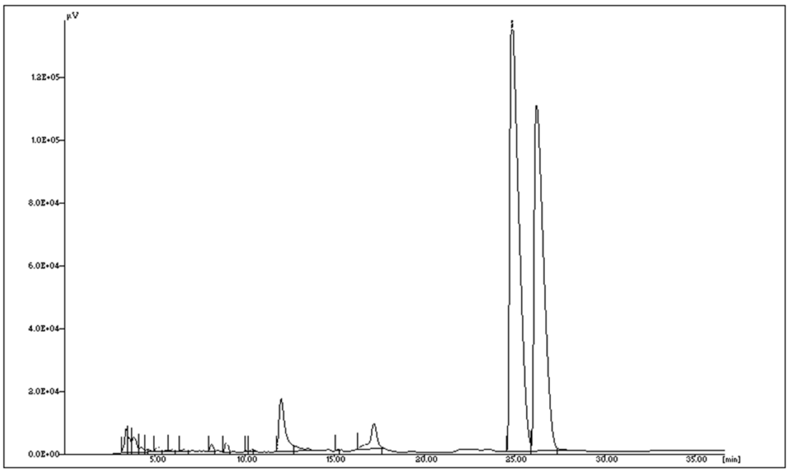

Peak Name	Rt (min)	Area	Factor
Benzylmethylsulfide	4.582	7591.61	1.000
(S)-benzyl methyl sulfoxide	24.817	4396125.60	1.000
(R)-benzyl methyl sulfoxide	26.184	3645161.58	1.000

Figure S17. HPLC chromatogram of sulfoxidation of benzyl methyl sulfide (**3**) catalyzed by HT21-AL1.

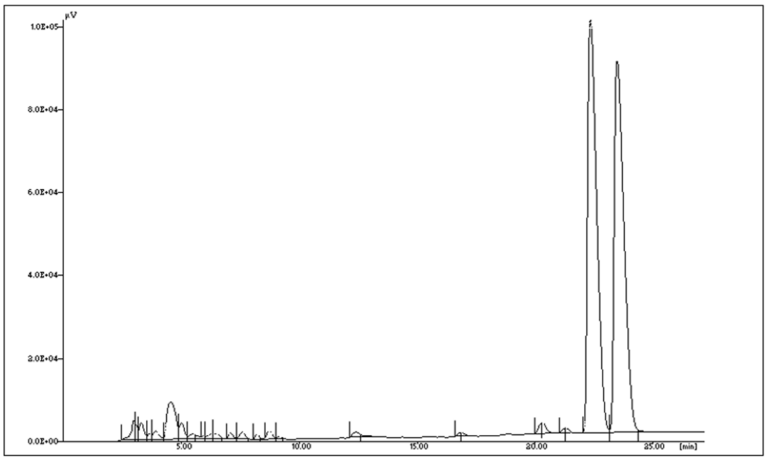

Peak Name	Rt (min)	Area	Factor
Benzylmethylsulfide	4.778	644869.49	1.000
(S)-benzyl methyl sulfoxide	23.574	3066356.35	1.000
(R)-benzyl methyl sulfoxide	24.798	2815369.48	1.000

Figure S18. HPLC chromatogram of sulfoxidation of benzyl methyl sulfide (**3**) catalyzed by HT21-AL2.

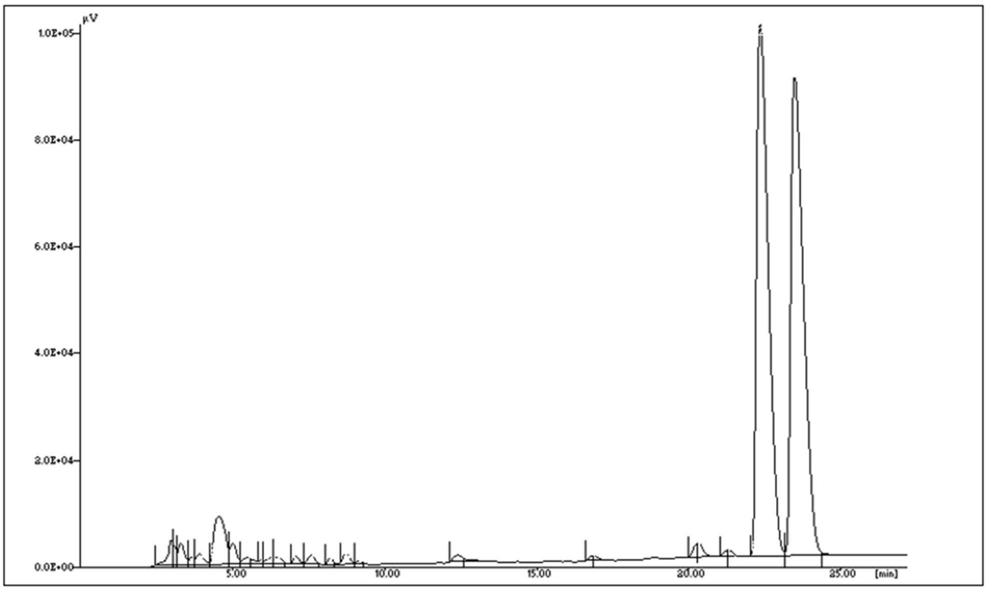

Peak Name	Rt (min)	Area	Factor
Benzylmethylsulfide	4.537	20.9431.75	1.000
(S)-benzyl methyl sulfoxide	22.324	2394170.15	1.000
(R)-benzyl methyl sulfoxide	23.455	2352936.15	1.000

Figure S19. HPLC chromatogram of sulfoxidation of benzyl methyl sulfide (**3**) catalyzed by HT21-L1.

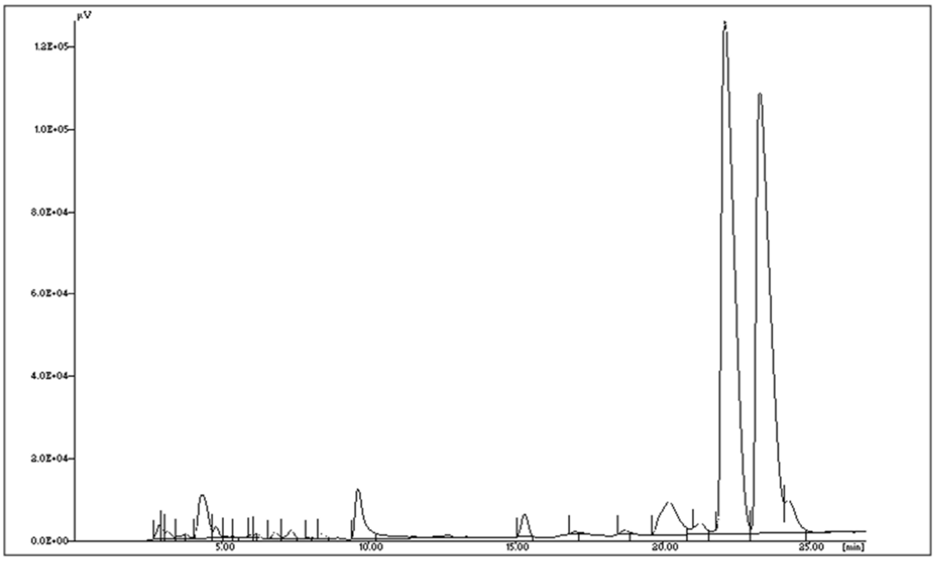

Peak Name	Rt (min)	Area	Factor
Benzylmethylsulfide	4.317	209468.23	1.000
(S)-benzyl methyl sulfoxide	22.112	3424187.22	1.000
(R)-benzyl methyl sulfoxide	23.297	3233374.54	1.000

Figure S20. HPLC chromatogram of sulfoxidation of benzyl methyl sulfide (**3**) catalyzed by HT21-L2.

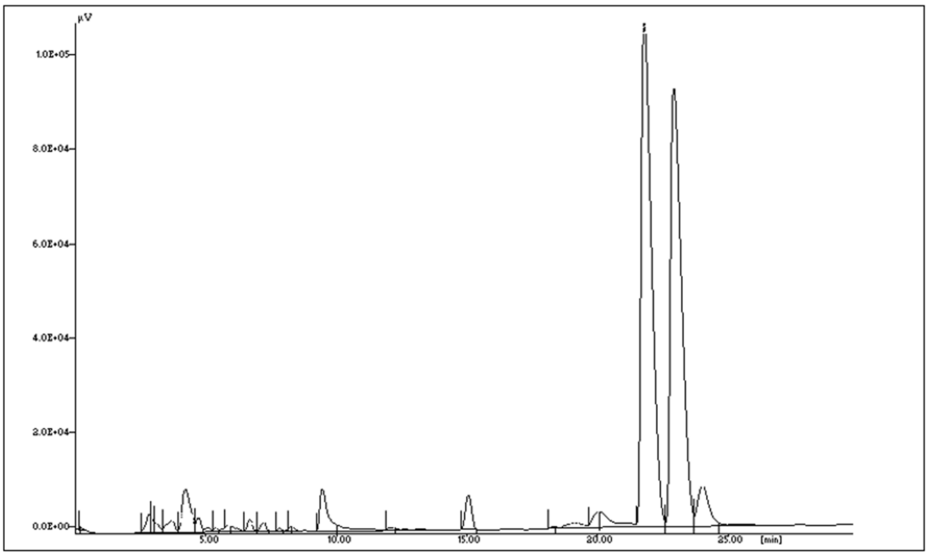

Peak Name	Rt (min)	Area	Factor
Benzylmethylsulfide	4.191	174948.27	1.000
(S)-benzyl methyl sulfoxide	21.777	2657932.17	1.000
(R)-benzyl methyl sulfoxide	22.904	2478253.00	1.000

Figure S21. HPLC chromatogram of sulfoxidation of benzyl methyl sulfide (**3**) catalyzed by HT21-B1.

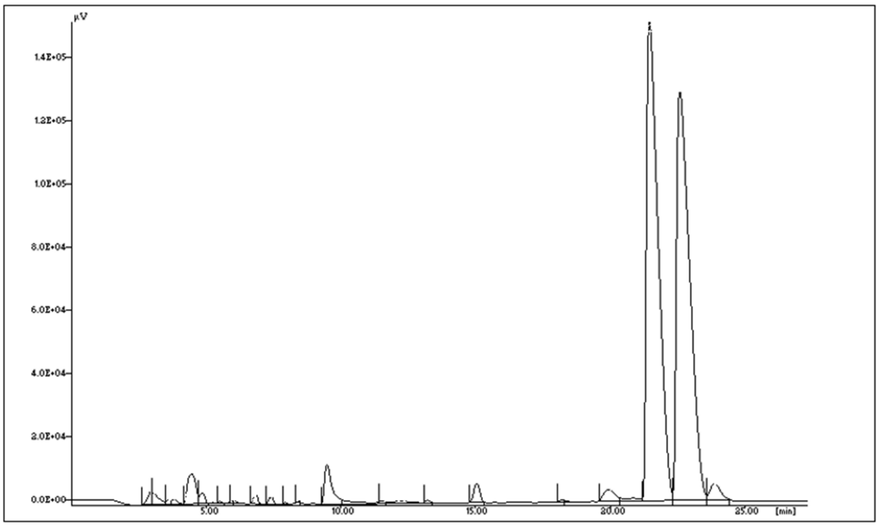

Peak Name	Rt (min)	Area	Factor
Benzylmethylsulfide	4.449	188206.91	1.000
(S)-benzyl methyl sulfoxide	21.443	4185675.30	1.000
(R)-benzyl methyl sulfoxide	22.568	4038493.76	1.000

Figure S22. HPLC chromatogram of sulfoxidation of thioanisole (**1**) with Cu**L** and without HT21.

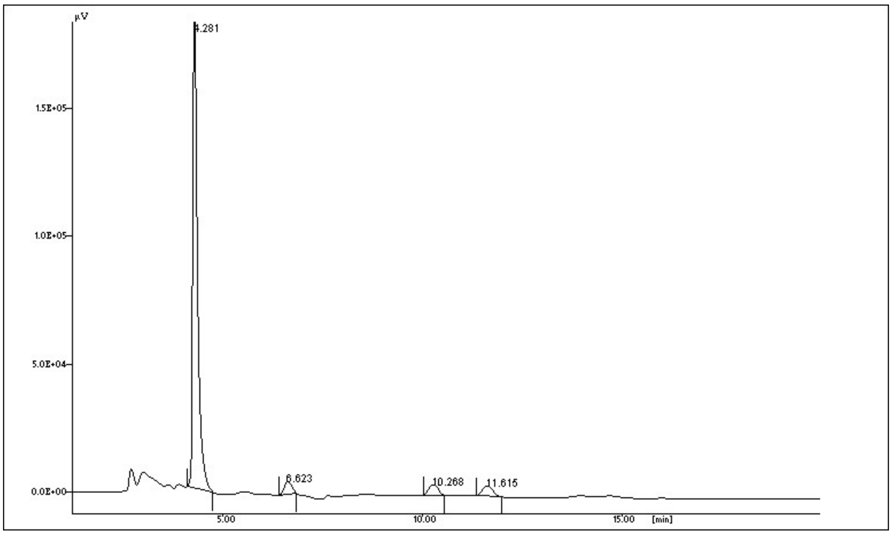

Peak Name	Rt (min)	Area	Factor
Thioanisole	4.281	1245084.20	1.000
(R)-methylphenyl sulfoxide	10.268	58701.125	1.000
(S)-methylphenyl sulfoxide	11.615	67944.935	1.000

Figure S23. HPLC chromatogram of sulfoxidation of thioanisole (**1**) without HT21 and Cu**L**.

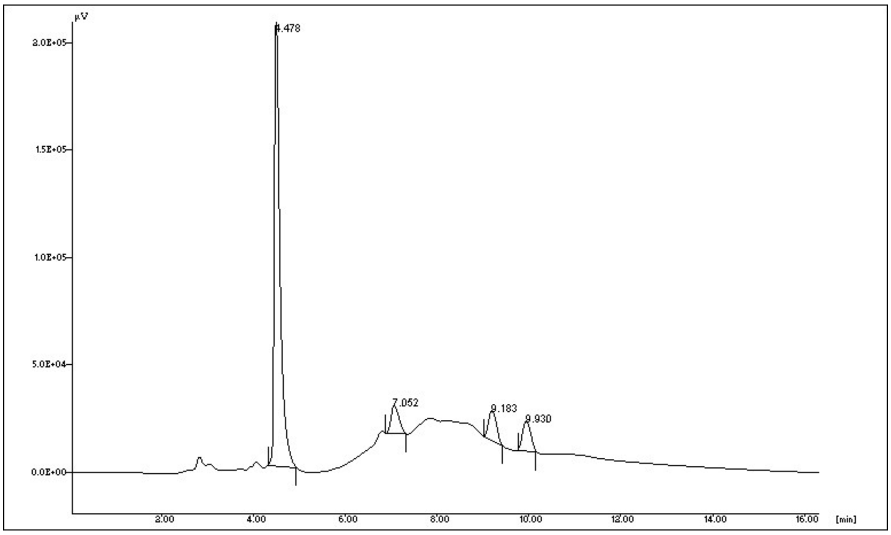

Peak Name	Rt (min)	Area	Factor
Thioanisole	4.478	1502455.73	1.000
(R)-methylphenyl sulfoxide	none	-	-
(S)-methylphenyl sulfoxide	none	-	-

Figure S24. ^{1}H NMR spectrum of methyl phenyl sulfoxide (**2**) (CD_3OD, 400 MHz).

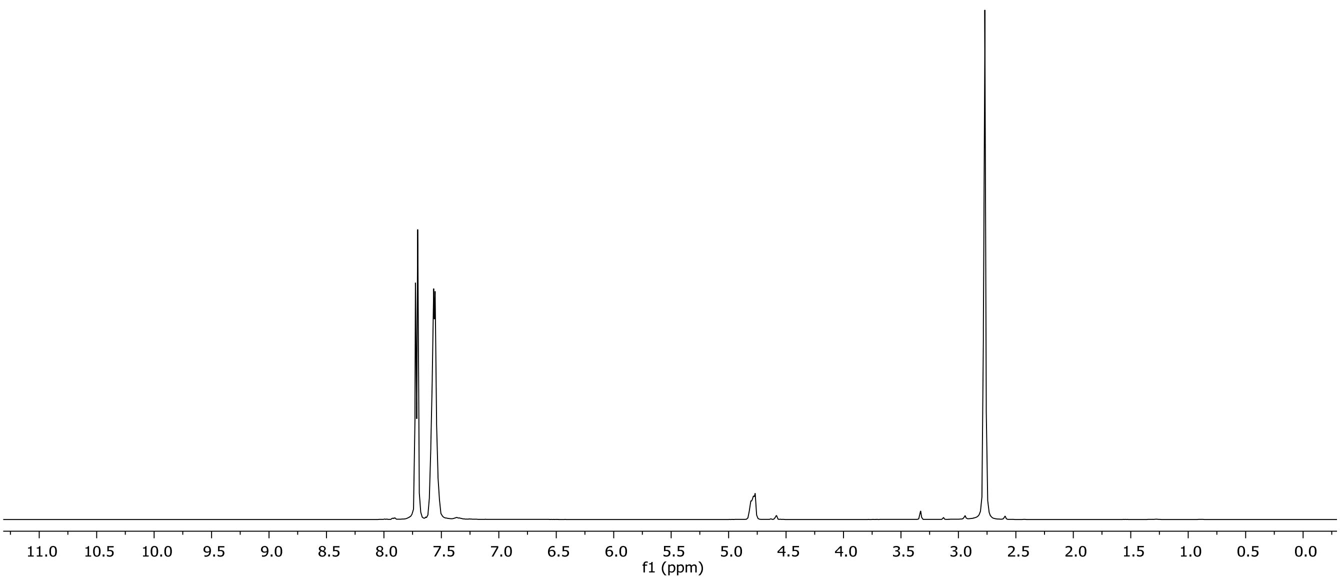

Figure S25. ^{13}C NMR spectrum of methyl phenyl sulfoxide (**2**) (CD_3OD, 100 MHz).

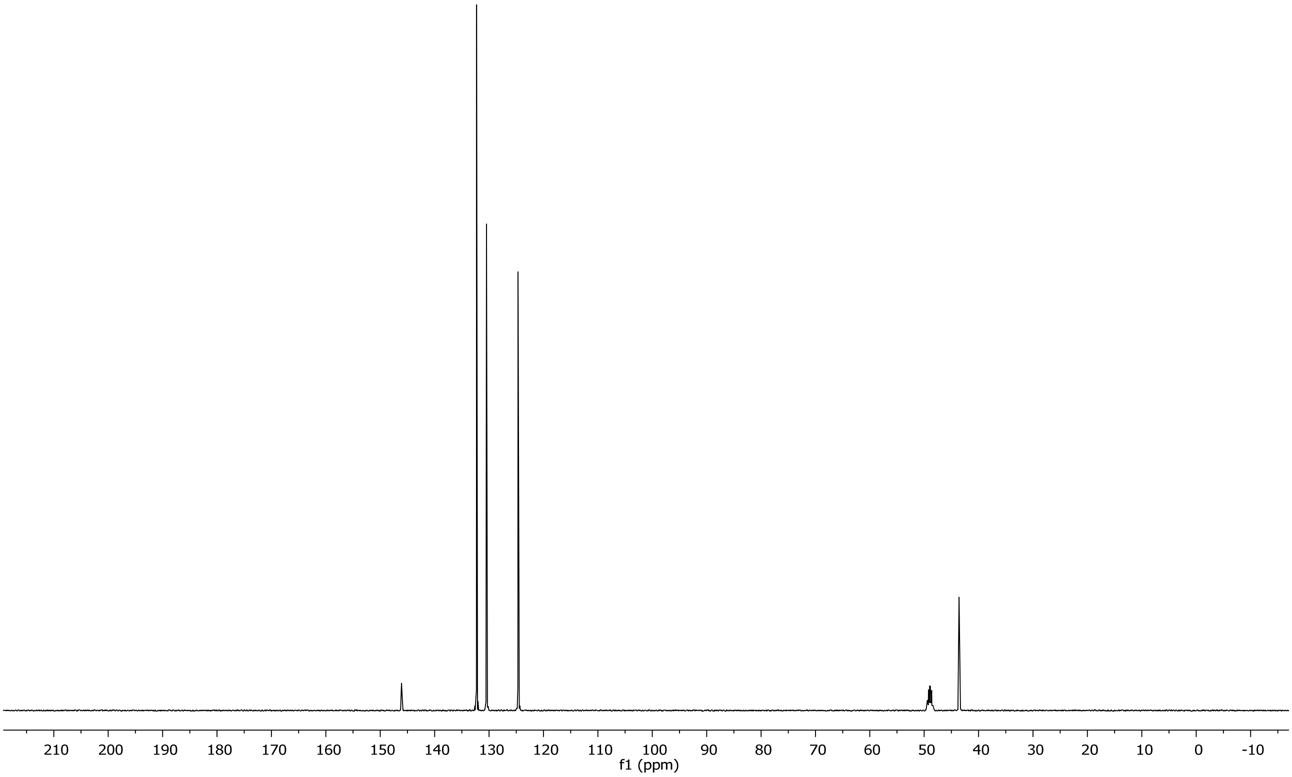

Figure S26. ESI-MS spectrum of methyl phenyl sulfoxide (**2**) $[M+H]^+$.

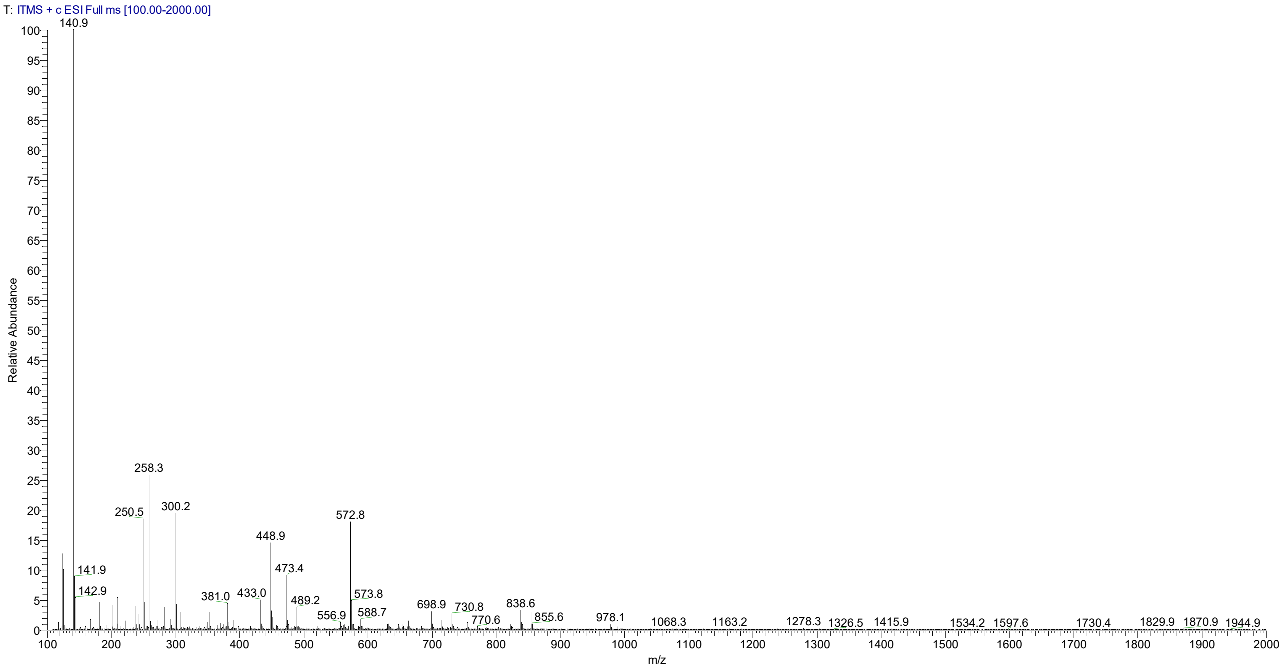

Figure S27. ^1H NMR spectrum of benzyl methyl sulfoxide (**4**) ($CDCl_3$, 400 MHz).

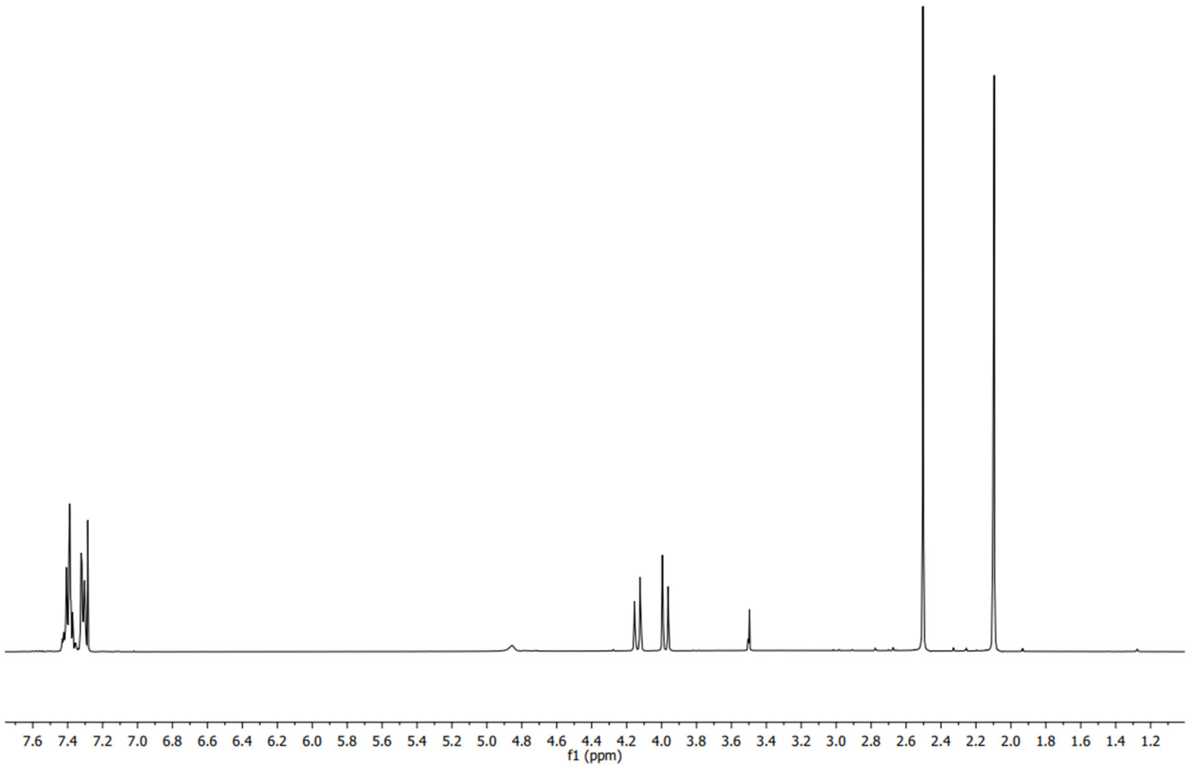

Supplement: Supplementary file 1 [file molecules-31-00442-s001.zip › molecules-4067420-supplementary.pdf]
